# International society of sports nutrition position stand: Beta-Alanine

**DOI:** 10.1186/s12970-015-0090-y

**Published:** 2015-07-15

**Authors:** Eric T. Trexler, Abbie E. Smith-Ryan, Jeffrey R. Stout, Jay R. Hoffman, Colin D. Wilborn, Craig Sale, Richard B. Kreider, Ralf Jäger, Conrad P. Earnest, Laurent Bannock, Bill Campbell, Douglas Kalman, Tim N. Ziegenfuss, Jose Antonio

**Affiliations:** Applied Physiology Laboratory, Department of Exercise and Sport Science, University of North Carolina, Chapel Hill, NC USA; Department of Sport and Exercise Science, University of Central Florida, Orlando, FL USA; Human Performance Laboratory, Department of Exercise Science, University of Mary Hardin-Baylor, Belton, TX USA; Health and Performance Enhancement Research Centre, Department of Sport Science, Nottingham Trent University, Nottingham, UK; Exercise & Sport Nutrition Lab, Department of Health & Kinesiology, Texas A&M University, College Station, TX USA; Increnovo LLC, 2138 E Lafayette Pl, Milwaukee, WI USA; Nutrabolt International, Bryan, TX USA; Guru Performance LTD, London, UK; Performance & Physique Enhancement Laboratory, University of South Florida, Tampa, FL USA; Department of Nutrition & Endocrinology, Miami Research Associates, QPS-MRA, Miami, FL USA; The Center for Applied Health Sciences, 4302 Allen Rd, STE 120 Stow, OH USA; Exercise and Sports Science, Nova Southeastern University, Davie, FL USA

## Abstract

The International Society of Sports Nutrition (ISSN) provides an objective and critical review of the mechanisms and use of beta-alanine supplementation. Based on the current available literature, the conclusions of the ISSN are as follows: 1) Four weeks of beta-alanine supplementation (4–6 g daily) significantly augments muscle carnosine concentrations, thereby acting as an intracellular pH buffer; 2) Beta-alanine supplementation currently appears to be safe in healthy populations at recommended doses; 3) The only reported side effect is paraesthesia (tingling), but studies indicate this can be attenuated by using divided lower doses (1.6 g) or using a sustained-release formula; 4) Daily supplementation with 4 to 6 g of beta-alanine for at least 2 to 4 weeks has been shown to improve exercise performance, with more pronounced effects in open end-point tasks/time trials lasting 1 to 4 min in duration; 5) Beta-alanine attenuates neuromuscular fatigue, particularly in older subjects, and preliminary evidence indicates that beta-alanine may improve tactical performance; 6) Combining beta-alanine with other single or multi-ingredient supplements may be advantageous when supplementation of beta-alanine is high enough (4–6 g daily) and long enough (minimum 4 weeks); 7) More research is needed to determine the effects of beta-alanine on strength, endurance performance beyond 25 min in duration, and other health-related benefits associated with carnosine.

## Introduction

Beta-alanine is a non-proteogenic amino acid that is produced endogenously in the liver. In addition, humans acquire beta-alanine through the consumption of foods such as poultry and meat. By itself, the ergogenic properties of beta-alanine are limited; however, beta-alanine has been identified as the rate-limiting precursor to carnosine synthesis [1, 2], and has been consistently shown to increase levels of carnosine in human skeletal muscle. Doses of 4 to 6 g/day of beta-alanine have been shown to increase muscle carnosine concentrations by up to 64 % after 4 weeks [1], and up to 80 % after 10 weeks [3]. Baguet et al. [4] demonstrated that individuals vary in the magnitude of response to 5 to 6 weeks of beta-alanine supplementation (4.8 g/day), with high responders increasing muscle carnosine concentrations by an average of 55 %, and low responders increasing by an average of only 15 %. The difference between high and low responders seems, at least in part, to be related to baseline muscle carnosine content and muscle fiber composition [5].

While evidence suggests that athletes engaged in resistance training and high-intensity exercise have higher concentrations of muscle carnosine [6, 7], longitudinal training studies have demonstrated equivocal changes in intramuscular carnosine [8–11]. The variability of increases in carnosine appears to be reflective of baseline levels, with vegetarians having greater increases in carnosine concentrations compared to carnivores. In humans, muscle carnosine contents generally range from 10 – 40 mmol/kg dry weight [5, 6, 12] with average values around 20–30 mmol/kg dry weight [5, 13–15], although these contents can be influenced by a number of factors. Carnosine concentrations tend to be higher in males compared to females [15], and in fast-twitch compared to slow-twitch muscle fibers [16–18]. Carnosine concentrations may also decline with age and is most likely influenced by habitual dietary intake of carnosine-containing foods (e.g. beef, pork, poultry, fish, etc.) [5, 14].

Despite this, beta-alanine supplementation will still increase carnosine concentrations, regardless of low or high baseline levels [19, 20], with no upper limit for muscle carnosine concentrations having yet been identified. While cross-sectional studies have shown higher baseline carnosine contents in the *gastrocnemius* muscle of sprinters [7] and resistance-trained athletes [6] versus their untrained counterparts, beta-alanine supplementation has also been shown to increase muscle carnosine in both trained [20] and untrained [1] populations. A recent study by Bex et al. [21] suggests that increases in whole muscle carnosine concentrations may be slightly higher in trained athletes compared to non-athletes supplementing with beta-alanine, but more research is needed to replicate this finding and account for potential differences in single muscle fiber concentrations. Much of the research evaluating increases in muscle carnosine has been performed in young males, but evidence also suggests that beta-alanine supplementation is effective in females [22, 23] and the elderly [24].

Over the past ten years, beta-alanine has grown to become one of the most popular sports nutrition ingredients. Although relatively new, with the first human study published in 2006, beta-alanine use and formulation has expanded into nearly every pre-workout formula on the market, and a number of daily and recovery formulas. In summary, the purpose of the International Society of Sports Nutrition Position Stand is to provide a critical review on the effects of beta-alanine and thus provide reasonable guidelines for its use as an ergogenic aid. This Position Stand is presented as a general review of literature, including a relative effects analysis to evaluate performance effects.

### Mechanism of action

Carnosine (β-Alanyl-L-histidine) is a naturally occurring dipeptide with numerous potential physiological functions and is formed by combining its constituent amino acids, L-histidine and beta-alanine, with the assistance of the enzyme carnosine synthetase. Carnosine is predominantly stored within skeletal muscle, and can vary widely between species [16]. Carnosinase, the enzyme that catalyzes the breakdown of carnosine, is present in serum and various tissues in humans, but is absent in skeletal muscle [25] and many animals. It is important to note that carnosinase is not present in most non-primate mammals [26], which must be considered when evaluating carnosine supplementation and data obtained from animal models. Therefore, oral carnosine supplementation is an inefficient method of augmenting muscle carnosine levels in humans, as ingested carnosine is ultimately metabolized before reaching skeletal muscle [27].

Carnosine’s role as an intracellular proton buffer was first identified by Severin et al. in 1953 [28], who demonstrated that the absence of carnosine resulted in more rapid fatigue and acidosis. By virtue of a pKa of 6.83 and high concentrations in muscle [29], carnosine has been shown to be more effective at sequestering protons than either bicarbonate (pKa 6.3) or inorganic phosphate (pKa 7.2), the other two major physio-chemical buffers, over the physiological pH range. With respect to carnosine’s structure, nitrogen atoms on the imidazole ring can readily accept a proton at physiological pH, and therefore it has been suggested that carnosine buffering precedes involvement of the bicarbonate buffering system during exercise [30]. Preliminary estimates of what contribution carnosine may play in buffering suggested as much as 40 % of the buffering capacity of muscle [31] when evaluated in animals; more recent research in humans has indicated the contribution may be as low as 7 % [15]. More evidence documenting the contribution of carnosine in muscle buffering is needed to further identify its role in exercise performance. Nonetheless, beta-alanine supplementation has been shown to increase muscle carnosine concentrations [1, 3] and attenuate exercise-induced reductions in pH [32], supporting the concept that carnosine plays a significant role in buffering exercise-induced acidosis.

The potential physiological roles of carnosine extend beyond its function as a proton buffer. Previous research has suggested that reactive oxygen species (ROS), which are produced at an elevated rate during exercise [33], may contribute to muscle fatigue and exercise-induced muscle damage under certain circumstances [34, 35]. Carnosine has been shown to act as an antioxidant by scavenging free radicals and singlet oxygen [36, 37], thereby reducing oxidative stress. Carnosine can further reduce oxidative stress by chelating transition metals, such as copper and iron [37]. In doing so, these transition metals are prevented from reacting with peroxides in the Fenton reaction, which results in the production of free radicals. Carnosine is abundant in human skeletal muscle, and may influence these contributors to fatigue and oxidative stress by buffering excess protons [28], scavenging free radicals [36, 37], and chelating transition metals [37]. As the rate-limiting precursor to carnosine synthesis, beta-alanine supplementation has been shown to consistently elevate carnosine in a variety of populations, and may therefore improve performance during high-intensity exercise and/or enhance the quality of training in athletes participating in strength and power sports [38].

- Beta-alanine works by enhancing muscle carnosine concentrations.

### Supplementation strategies

The supplementation strategy for beta-alanine is important to maximize its effects. To date, research suggests that beta-alanine requires a chronic loading dose of 4 to 6 g daily in divided doses of 2 g or less, for a minimum of two weeks (which results in a 20-30 % increase in muscle carnosine concentrations) [4], with greater benefits seen after 4 weeks (40-60 % increase) [19, 39]. To increase muscle carnosine, a larger dose of 6 g, divided into 4 equal doses would be more advantageous. Additionally, if supplementing with a non-time release version, consuming a total daily dose of 6 g would be important for augmenting muscle carnosine [40]. Single large boluses of beta-alanine have been shown to induce paraesthesia (i.e. tingling), and have not been effective for performance outcomes likely due to strong paraesthesia, rapid changes in pH, higher excretion rates, and inability to effectively load the muscle contents. Combining beta-alanine consumption with a meal during beta-alanine loading has also been shown to be effective for further augmenting muscle carnosine levels [41]. In addition, a recent meta-analysis [42] suggested that supplementation with a total ingestion of 179 g of beta-alanine (the average dose across all studies) resulted in a median performance improvement of 2.85 % compared with a placebo. Washout time, or time required for values to return to baseline, may vary between non-responders and responders, requiring 6 to 15 weeks to return to normal [4]. Despite these findings, the maximal concentration or retention of carnosine in human muscle is not well known; thus, we cannot yet provide information on the optimal loading or maintenance doses.

- A loading phase (~4 weeks) of beta-alanine supplementation is essential for increasing carnosine levels.

### Beta-alanine safety

Paraesthesia (i.e., tingling) is the most widely known side effect of beta-alanine and is commonly experienced in individuals consuming more than 800 mg of beta-alanine in a non-sustained release form [1]. It appears that the symptoms of paraesthesia are substantially reduced with the use of sustained-release formulations. In studies using the non-sustained release supplement, paraesthesia has generally been reported to disappear within 60 to 90 min following supplementation [40]. It is hypothesized that beta-alanine activates Mas-related genes (Mrg) [43], or sensory neuron specific G-protein coupled receptors. Specifically, MrgD, which is expressed in the dorsal root ganglion, terminates in the skin [44]. It is likely that activation of MrgD from beta-alanine results in paraesthesia on the skin. To date, there is no evidence to support that this tingling is harmful in any way. The paraesthesia side effect is typically experienced in the face, neck, and back of hands. Although not all individuals will experience paraesthesia, it is typically dose-dependent, with higher doses resulting in greater side effects. Recent data also suggests that males of Asian descent may experience a reduced effect, with Asian females experiencing greater paraesthesia [45]. Moreover, there is no known mechanism to explain why certain individuals may be predisposed to experiencing paraesthesia. Currently, there is no safety data on the long-term use of beta-alanine (i.e., > 1 year). However, due to the non-essential nature of this constituent (i.e., beta-alanine is produced endogenously), the likelihood of safety concerns are low.

A secondary effect of beta-alanine supplementation is a potential decrease in taurine concentrations. Beta-alanine and taurine share the same transporter (Tau-T) into skeletal muscle, with beta-alanine thereby inhibiting taurine uptake within the muscle [46]. In animal models, beta-alanine has been shown to decrease circulating taurine levels by about 50 % [47]. Interestingly, Harris et al. [1] reported that 4 weeks of beta-alanine supplementation (10–40 mg∙kg^−1^bw) resulted in an increase in plasma taurine concentration; however, there was no significant decrease in muscle taurine content. While taurine has a number of essential physiological functions, to date there is no human data to support decreases with beta-alanine supplementation. Additionally, when extrapolated to humans, the decrease in taurine would not be of physiological significance.

- Current, although limited information, suggests that beta-alanine is safe in healthy individuals at recommended doses.

### Consensus of findings

To gain a better consensus of published findings, this review includes an analysis of the relative effects (RE) of literature obtained from PubMed and Google Scholar databases. The primary search terms included *beta-alanine* AND *supplementation* AND *carnosine* AND *exercise.* The search was limited to articles published as of March 2015 and written in English. To construct figures, literature with similar outcome variables was reviewed to identify studies evaluating the effects of beta-alanine supplementation for (a) open-ended exercise tasks, such as time to exhaustion (TTE), (b) fixed end-point exercise such as time trials, or (c) indices of neuromuscular fatigue.

To graphically depict the RE of beta-alanine in in comparison to placebo, RE was calculated using the following equation [48, 49]:

$$ RE=\left(\frac{\left(\frac{{\mathrm{Post}}_{\mathrm{BA}}}{{ \Pr \mathrm{e}}_{\mathrm{BA}}}\right)\times 100}{\left(\frac{{\mathrm{Post}}_{\mathrm{Pl}}}{{ \Pr \mathrm{e}}_{\mathrm{Pl}}}\right)\times 100}\right)\times 100 $$

Where Pre_PL_is the pre-test value in the placebo group, Post_PL_ is the post-test value in the placebo group, Pre_BA_ is the pre-test value in the beta-alanine group, and Post_BA_ is the post-test value in the beta-alanine group.

**Fig. 1:**
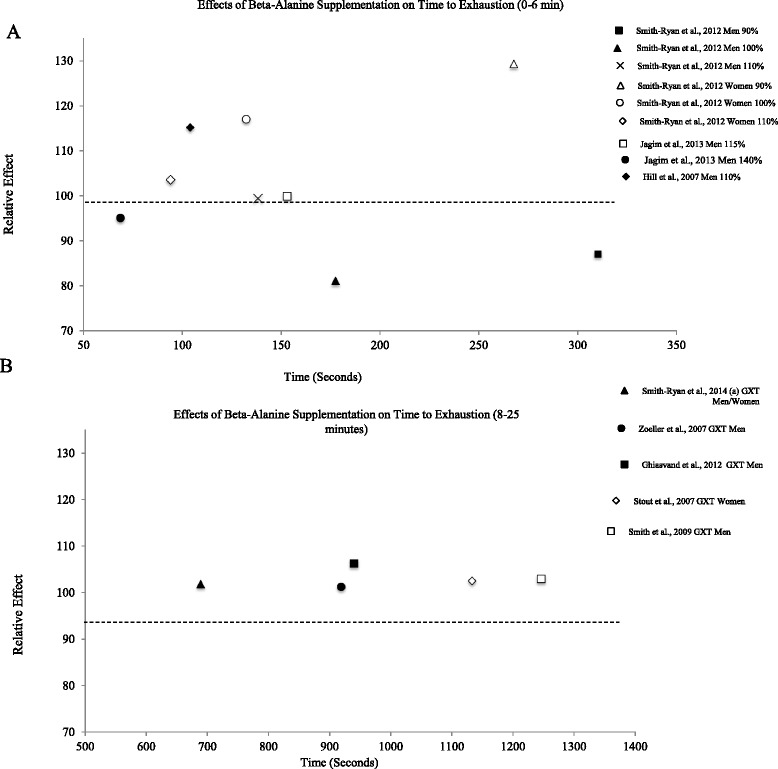
The relative effects of beta-alanine supplementation on time to exhaustion (TTE) lasting (A) 0–350 s (0–6 min) and (B) lasting 500–1400 s (8–25 min)

**Fig. 2:**
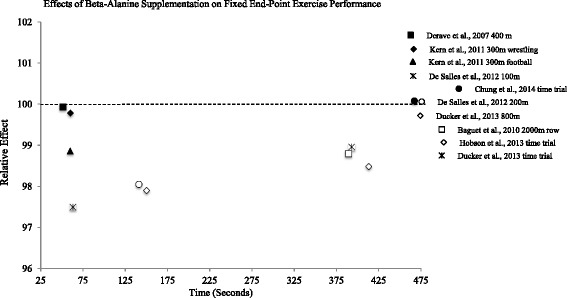
Relative effects of beta-alanine on time trial/fixed end-point exercise performance

**Fig. 3:**
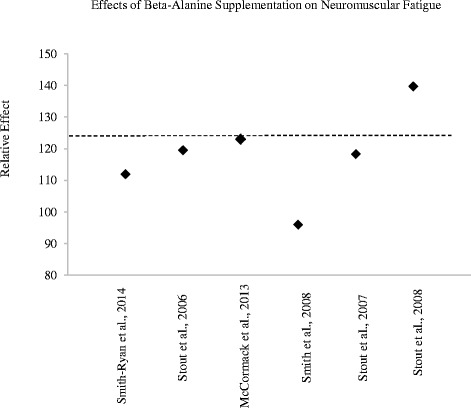
Relative effects of beta-alanine on neuromuscular fatigue (i.e. threshold/workload that can be sustained without fatigue)
- For time trial or fixed end-point data (Fig. 2), a relative effect of less than 100 demonstrates an ergogenic effect.

For Figures 1 and 3, an RE greater than 100 represents an increase or improvement in performance versus a placebo group. In Fig. 2, an RE less than 100 represents an improvement for fixed end-point tests, such as cycling time trials, where participants completed their work tasks faster.

- Relative effects were calculated to compare a number of studies on the same parameter.
- For time to exhaustion and neuromuscular fatigue (Figs. 1 and 3), a relative effect over 100 demonstrates an ergogenic effect of beta-alanine compared to placebo.

### Effects of Beta-alanine on exercise performance

It has been suggested that chronic beta-alanine supplementation improves high-intensity exercise performance by increasing muscle carnosine content, thereby enhancing intracellular proton buffering [50, 51]. Excess protons are also buffered independently of carnosine by a number of physicochemical buffering constituents; extracellular bicarbonate is the most relevant for increasing muscle buffering capacity [52], thereby acting to maintain intramuscular pH. As a result of augmented muscle buffering and mitigating H^+^ accumulation, beta-alanine has been suggested to be most beneficial in activities limited by acidosis, generally ranging from 2 to 4 min [25]. A collective view of the literature on anaerobic (0–4 min) and aerobic performance, neuromuscular fatigue, strength, and tactical challenges has been included.

#### Anaerobic exercise performance

The primary physiological mechanism associated with beta-alanine supplementation is most likely related to enhancing intracellular buffering capacity, consequently it has been hypothesized that beta-alanine supplementation would have ergogenic potential for activities that are primarily reliant on anaerobic metabolism. A meta-analysis on beta-alanine supplementation [42] indicated that supplementation improved exercise capacity in tasks lasting 60 to 240 s, but not in tasks lasting under 60 s in which acidosis is not likely the primary limiting factor. Additionally, literature evaluating repeated short-duration sprint tasks do not seem to demonstrate an effect: Sweeney et al. [53] reported no significant improvements in power output in repeated five-second bouts, and Derave et al. [20] did not report significant improvements in 400 m sprint time in response to beta-alanine supplementation (average sprint time = 51.3 s).

The effects of beta-alanine supplementation on time to exhaustion (TTE) are presented in Fig. 1 [3, 22, 54–60], with effects on fixed end-point exercise, such as races and time trials, presented in Fig. 2 [20, 61–67]. Similar to the results of Hobson et al. [42], the most pronounced effects of beta-alanine supplementation on TTE are seen in tasks under 270 s. For example, Hill et al. [3] reported marked improvements in cycling TTE at 110 % of maximal power output (average time = 104.1 s), resulting in a relative effect of 115.2, suggesting an improvement in performance (Fig. 1). A similar percentage increase (13-14 %) in cycling TTE for the beta-alanine groups was reported by Sale et al. [68] and Danaher et al. [69].

In a critical velocity test, Smith-Ryan et al. [56] showed large improvements in TTE for female participants running at 90 % and 100 % of the velocity at which VO_2_max was achieved (average time = 267.6 and 132.3 s), resulting in relative effects of 129.3 and 117.0, respectively. It should be noted that results are not entirely consistent, as relative effects below 100 are seen for anaerobic exercise tests between 1 to 4 min, as reported in Fig. 1. According to data from Jagim et al. [55], beta-alanine resulted in a relative effect of 95.1 for sprinting at 140 % of VO_2_max (Fig. 1). Further, data from Smith-Ryan et al. [56] indicated relative effects of 81.1 and 87.1 for male participants running at 100 % and 90 % of the velocity at which VO_2_max was achieved, respectively. In all three instances, relative effect calculations were influenced by substantial performance improvements in placebo groups ranging from 8 to 15 %.

In a recent meta-analysis, Hobson et al. [42] concluded that beta-alanine improved exercise capacity, or open end-point tests to volitional exhaustion, to a greater extent than fixed end-point exercise performance, such as race times or time trial performance. Relative effects for fixed-endpoint performance are displayed in Fig. 2. In agreement with Hobson et al. [42], relative effect values near 100 indicate modest effects of beta-alanine supplementation. Nonetheless, the three largest relative effects were observed in exercise bouts lasting 63.2-141.0 s [62, 63]. Taken together, research currently suggests that beta-alanine has the greatest potential to improve performance in high-intensity exercise lasting over 60 s, with more pronounced effects observed in open end-point exercise tasks taken to volitional exhaustion.

- Beta-alanine generally enhances high intensity exercise lasting over 60 s, with greater effects on open end point exercise bouts, such as time to exhaustion tasks*.*

#### Aerobic exercise performance

For exercise bouts lasting greater than four minutes, ATP demand is increasingly met via aerobic metabolic pathways. As such, it has been suggested that beta-alanine is not beneficial for exercise bouts lasting over 4 min. To the contrary, however, Hobson et al. [42] concluded that beta-alanine supplementation resulted in improvements of exercise tests of >4 min duration, when compared to the effect of a placebo, although the effect size calculated was smaller in comparison to exercise bouts lasting 1 to 4 min.

Research has demonstrated a modest benefit of beta-alanine supplementation on TTE in exercise tests over 4 min in duration (Fig. 1). In conjunction with 6 weeks of interval training, Smith et al. [59] demonstrated larger improvements in TTE in a graded exercise test with beta-alanine supplementation compared to placebo. Participants consuming a placebo improved TTE from 1128.7 s to 1299.6 s, whereas the beta-alanine group improved from 1168.2 s to 1386.7 s (RE = 103.1; Fig. 1). Similarly, Stout et al. [22] showed that participants supplementing with beta-alanine for 28 days improved TTE in a graded exercise test from 1117.6 s to 1146.7 s, while no improvement was shown in the placebo group (RE = 102.6; Fig. 1). In aerobic, open end-point exercise, beta-alanine appears to result in modest improvements that, nonetheless, could be meaningful in competitive athletics, such as running, cycling, etc.

Benefits have also been reported using fixed end-point exercise bouts lasting over 4 min (Fig. 2). Baguet et al. [61] showed that participants supplementing with beta-alanine performed a 2,000-m rowing time trial 4.3 s faster than the placebo group, despite being 0.3 s slower at baseline. While such results suggest modest benefits (RE = 98.8; Fig. 2), changes of this magnitude may be meaningful to competitive athletes. Similarly, Ducker et al. [64] showed beta-alanine to improve 2,000-m rowing performance by 2.9 s, resulting in a relative effect of 99.0.

Currently, limited research is available for exercise over 25 min in duration. In a graded exercise test, Van Thienen et al. [57] reported that eight weeks of beta-alanine supplementation (2–4 g∙day^−1^) was unable to improve TTE more than placebo. Although the beta-alanine group did improve TTE from 49.7 to 54.2 min, slightly larger improvements were observed in the placebo group (48.4 to 53.5; RE = 99.0), suggesting beta-alanine had limited effects. Chung et al. [70] investigated the effects of beta-alanine supplementation on one-hour time trial performance in trained cyclists. Although beta-alanine supplementation substantially increased muscle carnosine concentrations, both the beta-alanine and placebo groups saw performance decrements following six weeks of supplementation [70]. Overall, available research indicates that beta-alanine provides a modest benefit for exercise lasting up to approximately 25 min in duration. To date, research beyond this time frame is limited and does not demonstrate a consistent positive effect.

- Beta-alanine may improve exercise duration during tasks requiring a greater contribution from aerobic energy pathways

#### Neuromuscular fatigue

The physical working capacity at fatigue threshold (PWC_FT_) indicates the highest cycling power output that results in a non-significant increase in *vastus lateralis* muscle activation. This measurement is a validated and reliable method of determining the power output at which the onset of neuromuscular fatigue occurs [71], and has been used to determine the effects of beta-alanine supplementation on neuromuscular fatigue.

In 2006, Stout et al. [71] reported a 16.9 % improvement in PWC_FT_ in men after 28 days of beta-alanine supplementation (RE = 119.5; Fig. 3). Similar results were reported in female participants the following year (14.4 % improvement; RE = 118.2) [22]. During 6 weeks of high-intensity interval training, Smith et al. [72] showed a 20.4 % improvement in electromyographic fatigue threshold (EMG_FT_) in recreationally active participants supplementing with beta-alanine combined with interval training. Despite marked improvements, the relative effect calculated was below 100, as the group consuming a placebo improved by 25.5 % with interval training alone (RE = 95.9; Fig. 3). Using slightly different methodology to quantify neuromuscular fatigue, Smith-Ryan et al. [60] found a modest (5.6 %) improvement of physical working capacity at heart rate threshold in young men and women consuming beta-alanine (RE = 111.9). The effects of beta-alanine on neuromuscular fatigue appear to be more pronounced in longer studies utilizing older subjects. In a sample of older subjects (age = 70.7 ± 6.2 years), McCormack et al. [73] showed that fortifying a nutritional supplement with 1200 mg of beta-alanine improved PWC_FT_ in comparison to placebo after 12 weeks of supplementation (RE = 123.0). In a similar sample (age = 72.8 ± 11.1 year), Stout et al. [24] showed 90 days of beta-alanine supplementation resulted in a 37.3 % improvement in PWC_FT_. Collectively, the evidence suggests that beta-alanine supplementation attenuates neuromuscular fatigue, particularly in older subjects. Improvements in fatigue threshold may be augmented with concurrent participation in high-intensity interval training.

- Beta-alanine attenuates neuromuscular fatigue, particularly in older subjects.

#### Strength outcomes

Studies investigating the effects of beta-alanine on strength outcomes have reported mixed findings. While short-term (30 days) studies by Hoffman et al. [38, 74] did not show statistically significant improvements in performance, supplementation was shown to increase training volume and reduced subjective ratings of fatigue. In a similar length study (4 weeks), Derave et al. [20] showed beta-alanine supplementation increased muscle carnosine content and attenuated fatigue in five sets of 30 maximal dynamic knee extensions, while isometric endurance was unaffected. In contrast, Sale et al. [75] demonstrated a significant improvement in isometric endurance following 4 weeks of supplementation.

It has been hypothesized that the documented improvements in training volume and fatigue may translate to meaningful changes over prolonged interventions. Despite improvements from baseline testing, Kern and Robinson [66] did not show eight weeks of beta-alanine supplementation to significantly improve flexed arm hang performance in wrestlers or football players compared to placebo. In a 10-week intervention, Kendrick et al. [8] showed significant improvements in isokinetic force production, whole body strength, arm curl repetitions to fatigue, and body composition, but with no difference between the beta-alanine and placebo groups. Finally, Hoffman et al. [76] investigated the effects of creatine monohydrate, creatine + beta-alanine, or placebo in conjunction with ten weeks of training. Compared to placebo, both creatine and creatine + beta-alanine significantly improved squat 1RM, bench press 1RM, and weekly squat intensity. Only creatine + beta-alanine improved body composition and weekly training volume for squat and bench press, but differences were not significantly greater than creatine alone. Collectively, the evidence suggests that beta-alanine may improve indices of training volume and fatigue for resistance exercise, but more long-term studies are needed to clarify potential effects on strength and body composition compared to placebo.

- Beta-alanine appears to increase training volume, however, current research does not indicate an additive benefit on strength gains during resistance training.

#### Tactical athletes

The training and duties of military personnel and other tactical athletes often consist of prolonged and rigorous exercise, resulting in reductions in physical and cognitive performance [77]. Beta-alanine supplementation may be advantageous in this population, potentially attenuating fatigue, enhancing neuromuscular performance, and reducing oxidative stress. In 2014, an expert panel published a review regarding the use of beta-alanine in military personnel [78]. The panel concluded that there was insufficient evidence to recommend the use of beta-alanine by military personnel [78]. More recently, the use of beta-alanine in tactical personnel was directly investigated by Hoffman et al. [77]. Soldiers involved in military training supplemented with either beta-alanine or placebo for 28 days, with researchers testing a number of outcomes pertaining to physical and cognitive performance. While cognitive performance was not affected, beta-alanine resulted in moderate improvements in peak power, marksmanship, and target engagement speed, compared to placebo [77]. A subsequent study by Hoffman et al. [79] showed beta-alanine to significantly increase muscle carnosine, cognitive function, and performance on a test simulating a 50-m casualty carry; however, beta-alanine did not improve performance in a 2.5 km run, one minute sprint, repeated sprints, or marksmanship. Recently, it was reported that beta-alanine had no significant effect on brain carnosine or cognitive function in non-tactical athletes [80]. While evidence in this population is scarce, it would appear that beta-alanine supplementation yields promising results for tasks relevant to tactical personnel. More research is needed to determine which tasks are consistently improved with supplementation.

- Initial results in tactical athletes demonstrate a positive effect on military-specific tasks.

### Beta-alanine combined with other sports supplements

The combined effects of beta-alanine with other ergogenic aids, such as sodium bicarbonate, creatine, and multi-ingredient pre-workout formulas, have gained popularity. Due to the potential positive effects of beta-alanine during high-intensity exercise, it has been hypothesized that combining it with other ergogenic aids may further augment performance and proton buffering.

Sodium bicarbonate (SB) supplementation has been shown to acutely increase bicarbonate levels, blood pH, and high-intensity exercise performance [81], prompting interest in combined supplementation with beta-alanine. Sale et al. [68] first examined the effects of this combination on exercise performance and showed that beta-alanine supplementation alone improved performance on a cycling test at 110 % of maximal power output, and that there was a 70 % probability of an additive effect of beta-alanine + SB compared to beta-alanine alone. Tobias et al. [82] investigated the effects of beta-alanine, SB, or the combination on repeated upper-body Wingate performance, separated by 3 min of rest. Both beta-alanine and SB improved mean power output, but the results for the beta-alanine + SB group were superior, but not significant, compared to either supplement alone. Despite non-significant differences between groups, authors of other studies have calculated the probability of an additive effect with combined beta-alanine and SB supplementation. In a 2,000-m rowing time trial, Hobson et al. [65] used magnitude-based inferences to determine that beta-alanine was very likely to improve time trial performance (96 % chance of positive effect), SB was likely to improve performance (87 % chance), and that adding acute SB supplementation to chronic beta-alanine consumption had a small, possibly beneficial affect compared to beta-alanine alone (64 % probability). In swimmers, de Salles Painelli et al. [62] showed a 71.8 % and 78.5 % probability of an additive effect on 100-m and 200-m sprints, when adding SB to beta-alanine supplementation. In contrast to these studies, other findings do not suggest a synergistic effect between beta-alanine and SB.

In a series of two repeated 100-m sprints in swimmers, Mero et al. [83] showed that SB supplementation alone attenuated increases in sprint time for the second sprint, but neither beta-alanine nor beta-alanine + SB resulted in significant improvements compared to placebo. Ducker et al. [84] investigated the efficacy of beta-alanine and SB in the context of a repeated sprint test consisting of three sets of 6 (18 total), 20-m sprints. Results demonstrated that SB supplementation improved performance more than placebo, beta-alanine, or a combination of beta-alanine and SB. Saunders et al. [85] had participants complete a repeated sprint protocol (five bouts of 6-s sprints) before, in the middle, and after a simulated football game in hypoxic conditions. Results indicated that neither beta-alanine, SB, nor beta-alanine plus SB improved performance on the sprint test. Bellinger et al. [86] showed that SB improved mean power output on a 4-min cycling test, but beta-alanine did not. While not statistically significant, subjects consuming beta-alanine + SB did improve power slightly more than those consuming SB alone, and 6 of 7 participants consuming beta-alanine saw an increase in average power output after additional supplementation with SB. It is also important to note that the protocols employed by Ducker et al. [84] and Saunders et al. [85] consisted of very short bouts (<7 s), in which proton buffering would not be the primary factor limiting performance.

Collectively, the body of literature suggests a modest additive effect when adding SB to beta-alanine supplementation in exercise bouts in which metabolic acidosis may be performance-limiting. While this additive benefit is not typically revealed with traditional statistical analyses, studies using magnitude-based inferences have suggested that a modest additive effect is likely to exist [62, 65, 68]. The studies reviewed have used supplement dosages ranging from 4.8-6.8 g/kg/day of beta-alanine for at least 28 days, and 0.3-0.5 g/kg of SB taken acutely. However, the only study to indicate a statistically significant synergistic effect of beta-alanine and SB [82] employed a unique dosing protocol for SB, providing daily doses of 0.5 g/kg/day for seven days, whereas other studies typically provide a dose of 0.3 g/kg acutely in the hours preceding the exercise bout. Individual responses to SB supplementation may vary, likely due to side effects including headache and gastrointestinal discomfort [68, 85, 87]. In terms of practical application, those wishing to combine beta-alanine and SB supplementation must carefully evaluate the dosage and timing with which SB is consumed and weigh the modest additive benefit against the risk of potentially ergolytic side effects.

Given the proton-buffering capacity of muscle carnosine [51], beta-alanine is most commonly purported to improve performance in exercise of high enough intensity to induce intramuscular acidosis. Creatine supplementation has been consistently shown to improve high-intensity exercise performance, primarily by increasing phosphorylcreatine and adenosine triphosphate (ATP) availability [88]. The first study investigating co-ingestion of these ingredients was reported in a published abstract by Harris et al. [89], finding that power output in a 4-min cycling test was improved more by creatine + beta-alanine than creatine alone. Similarly, Hoffman et al. [76] showed greater improvements in lean mass, fat mass, and strength in creatine + beta-alanine compared to creatine alone. Notably, these studies did not include a treatment arm ingesting beta-alanine alone. Zoeller et al. [58] investigated the effects of beta-alanine and creatine on performance on a graded, maximal exercise test on a cycle ergometer. No significant group effects were shown (creatine, beta-alanine, creatine + beta-alanine, or PL), but authors noted that the creatine + beta-alanine did have significant within-group improvements for 5 of the 8 outcomes measured, compared to only one in the beta-alanine group and two in the creatine group. Stout et al. [71] showed that beta-alanine and creatine + beta-alanine improved PWC_FT_ compared to creatine and PLA. There was no evidence of a synergistic effect on this outcome, as CRE + beta-alanine was not significantly different than beta-alanine alone. Kresta et al. [90] investigated both aerobic and anaerobic exercise outcomes. The creatine group trended toward an increase in VO_2_max, while the beta-alanine group trended toward an improvement in rate of fatigue on a series of two Wingate tests. However, no significant effects on performance were noted for any treatment arm, and results did not suggest a synergistic effect between creatine and beta-alanine.

Two studies have shown additive ergogenic effects when beta-alanine is combined with creatine supplementation [76, 89], but did not include a treatment group ingesting beta-alanine only. Other studies including a beta-alanine treatment arm have not demonstrated a synergistic effect between beta-alanine and creatine [71, 90]. Despite promising findings from initial studies [76, 89], more research is needed to evaluate potential synergy between creatine and beta-alanine supplementation. Based on the ergogenic mechanisms of each ingredient, performance improvements are more likely to occur in high-intensity bouts of exercise, and studies investigating exercise bouts over 15 min in duration have not shown beta-alanine + creatine to be significantly more effective than placebo [58, 90].

Multi-ingredient pre- and post-workout supplements have become increasingly popular, with formulations that include a number of purportedly ergogenic ingredients including creatine, caffeine, branched-chain amino acids, whey protein, nitric oxide precursors, and other isolated amino acids [91–98]. Such supplements are typically consumed once per day prior to training, with beta-alanine doses generally ranging from 2 to 4 g single boluses. When ingested acutely before exercise, previous studies have shown these multi-ingredient supplements to improve muscular endurance [92, 98], running time to exhaustion [91], and power output [98]. Some studies have documented improvements in subjective feelings of energy and focus [91, 92], while Gonzalez et al. [98] did not. When taken chronically for a period of 4 to 8 weeks, multi-ingredient pre-workout supplements have been shown to increase measures of strength [93, 94, 97], power output [96], and lean mass [93–95]. In contrast, Outlaw et al. [99] found no significant benefit for body composition, strength, or power output with ingestion of a multi-ingredient supplement versus placebo. While the supplement group tended to improve leg press strength to a greater degree than the placebo group, this difference was not statistically significant (p = 0.08). These discrepant findings may be attributed to the short duration of supplementation (8 days), or the substantial improvements in lean mass, strength, and peak power output displayed by the placebo group.

Overall, the body of literature suggests that acute and chronic ingestion of multi-ingredient pre-workout supplements can contribute to improvements in performance and body composition. It is difficult to attribute these ergogenic effects directly to beta-alanine, as multi-ingredient supplements include a wide range of ergogenic ingredients that may improve performance independently (e.g., caffeine, creatine, etc.). It typically takes a number of weeks (at least 2 weeks) for beta-alanine supplementation to yield meaningful increases in muscle carnosine content [3, 19]. As such, it is unlikely that beta-alanine is the primary ingredient improving performance outcomes in studies utilizing acute, one-time supplementation. In studies extending over 4 to 8 weeks, the likelihood of beta-alanine contributing to improvements in performance and indirect effects on body composition is greater. While it is difficult to determine the relative contributions of individual ingredients, research has demonstrated that multi-ingredient pre-workout supplements containing 2 to 4 g of beta-alanine are safe and efficacious when taken acutely, or chronically for up to 8 weeks.

- Co-ingestion of beta-alanine with sodium bicarbonate or creatine have modest additive ergogenic benefits; ingestion of beta-alanine as part of a multi-ingredient pre-workout product may be effective, if the supplementation period is sufficient to increase carnosine levels and the product is taken for at least 4 weeks.

### Health

Decades of literature support a potential for carnosine to influence some mechanisms related to health including antioxidant properties, anti-aging, immune enhancing, and neurotransmitter actions. However, the majority of these health benefits have been explored *in vitro* and in animal models. Carnosine is widely considered an important anti-glycating agent that serves to prevent reactions that threaten to impact the structure and function of proteins in the body. Advanced glycation end products are associated with the aging process and diabetic complications, but carnosine is thought to reduce the formation of these end products [100, 101]. Previous research has also indicated that carnosine acts as a “sacrificial peptide,” reacting with carbonyl groups of aldehydes, ketones, and proteins to prevent damage to proteins [102, 103].

Carnosine is also known to be an antioxidant that is capable of preventing the accumulation of oxidized products derived from lipid components of biological membranes [104, 105]. The antioxidant mechanism of carnosine has been postulated to be due to metal chelation or free radical scavenging [106]. The combination of histidine-containing compounds, such as carnosine, at near physiological concentrations, have resulted in synergistic antioxidant activity [37]. Minimal data in humans exists regarding the potential antioxidant effect of increasing muscle carnosine *vis-a-vis* beta-alanine. Initial research suggests that beta-alanine may effectively reduce lipid peroxidation and mitigate accumulation of free radicals when combined with aerobic exercise in men and women [107, 108]. Future research evaluating potential anti-aging effects and the impact of potential antioxidant properties in humans would be important to explore, especially due to the positive effects beta-alanine has shown in older populations [24, 73]

- Beta-alanine may act as an antioxidant.

### Remaining questions

It is widely accepted that as a result of increases in muscle carnosine concentration, the primary mechanism driving enhanced performance is the improvement in H^+^ buffering within skeletal muscle. Interestingly, humans also have carnosine within the brain, eye, and heart tissue [37, 109]. Therefore some initial data has explored the neuronal effects of carnosine [80, 110], as well as potential effects on cardiac tissue and heart rate [60]. Future research exploring the effects of beta-alanine to induce changes in carnosine concentrations in these tissues would be beneficial, as well as explorations of potential physiological effects in humans. An additional potential function of carnosine has been linked to improvements in calcium sensitivity in muscle fibers [111, 112]. As a result of improved calcium sensitivity, there may be a direct impact on muscular performance. This mechanism has not yet been fully explored in humans. One recent paper by Hannah et al. [113] suggests that an improvement in calcium kinetics is not the mechanism by which beta-alanine influences performance. Future studies should further explore this mechanism. Lastly, there is a need for long-term safety data on beta-alanine supplementation as well as more information on potential benefits in special populations such as elderly and tactical athletes.

### Summary & recommendations

- Four weeks of beta-alanine supplementation (4–6 g daily) significantly augments muscle carnosine concentrations, thereby acting as an intracellular pH buffer.
- Beta-alanine supplementation currently appears to be safe in healthy populations at recommended doses.
- The only reported side effect is paraesthesia (i.e., tingling) but studies indicate this can be attenuated by using divided lower doses (1.6 g) or using a sustained-release formula.
- Daily supplementation with 4 to 6 g of beta-alanine for at least 2 to 4 weeks has been shown to improve exercise performance, with more pronounced effects in open end-point tasks/time trials lasting 1 to 4 min in duration.
- Beta-alanine attenuates neuromuscular fatigue, particularly in older subjects, and preliminary evidence indicates that beta-alanine may improve tactical performance.
- Combining beta-alanine with other single or multi-ingredient supplements may be advantageous when the dose of beta-alanine is sufficient (i.e., 4–6 g daily) and the treatment duration is at least 4 weeks.
- More research is needed to determine the effects of beta-alanine on strength, endurance performance beyond 25 min in duration, and other health-related benefits associated with carnosine.

## References

[1] Harris RC, Tallon MJ, Dunnett M, Boobis L, Coakley J, Kim HJ (2006). The absorption of orally supplied beta-alanine and its effect on muscle carnosine synthesis in human vastus lateralis. Amino Acids.

[2] Dunnett M, Harris RC (1999). Influence of oral beta-alanine and L-histidine supplementation on the carnosine content of the gluteus medius. Equine Vet J Suppl.

[3] Hill CA, Harris RC, Kim HJ, Harris BD, Sale C, Boobis LH (2007). Influence of beta-alanine supplementation on skeletal muscle carnosine concentrations and high intensity cycling capacity. Amino Acids.

[4] Baguet A, Reyngoudt H, Pottier A, Everaert I, Callens S, Achten E (2009). Carnosine loading and washout in human skeletal muscles. J Appl Physiol.

[5] Harris RC, Jones G, Hill CH, Kendrick IP, Boobis L, Kim CK (2007). The carnosine content of vastus lateralis in vegetarians and omnivores. FASEB J.

[6] Tallon MJ, Harris RC, Boobis LH, Fallowfield JL, Wise JA (2005). The carnosine content of vastus lateralis is elevated in resistance-trained bodybuilders. J Strength Cond Res.

[7] Baguet A, Everaert I, Hespel P, Petrovic M, Achten E, Derave W (2011). A new method for non-invasive estimation of human muscle fiber type composition. PLoS One.

[8] Kendrick IP, Harris RC, Kim HJ, Kim CK, Dang VH, Lam TQ (2008). The effects of 10 weeks of resistance training combined with beta-alanine supplementation on whole body strength, force production, muscular endurance and body composition. Amino Acids.

[9] Kendrick IP, Kim HJ, Harris RC, Kim CK, Dang VH, Lam TQ (2009). The effect of 4 weeks beta-alanine supplementation and isokinetic training on carnosine concentrations in type I and II human skeletal muscle fibres. Eur J Appl Physiol.

[10] Mannion AF, Jakeman PM, Willan PL (1994). Effects of isokinetic training of the knee extensors on high-intensity exercise performance and skeletal muscle buffering. Eur J Appl Physiol Occup Physiol.

[11] Suzuki Y, Ito O, Takahashi H, Takamatsu K (2004). The effect of sprint training on skeletal muscle carnosine in humans. Int J Sport Health Sci.

[12] Boldyrev AA, Aldini G, Derave W (2013). Physiology and pathophysiology of carnosine. Physiol Rev.

[13] Derave W, Everaert I, Beeckman S, Baguet A (2010). Muscle carnosine metabolism and beta-alanine supplementation in relation to exercise and training. Sports Med.

[14] Everaert I, Mooyaart A, Baguet A, Zutinic A, Baelde H, Achten E (2011). Vegetarianism, female gender and increasing age, but not CNDP1 genotype, are associated with reduced muscle carnosine levels in humans. Amino Acids.

[15] Mannion AF, Jakeman PM, Dunnett M, Harris RC, Willan PL (1992). Carnosine and anserine concentrations in the quadriceps femoris muscle of healthy humans. Eur J Appl Physiol Occup Physiol.

[16] Abe H (2000). Role of histidine-related compounds as intracellular proton buffering constituents in vertebrate muscle. Biochemistry (Mosc).

[17] Harris RC, Dunnett M, Greenhaff PL (1998). Carnosine and taurine contents in individual fibres of human vastus lateralis muscle. J Sports Sci.

[18] Dunnett M, Harris RC (1997). High-performance liquid chromatographic determination of imidazole dipeptides, histidine, 1-methylhistidine and 3-methylhistidine in equine and camel muscle and individual muscle fibres. J Chromatogr B Biomed Sci Appl.

[19] Stellingwerff T, Anwander H, Egger A, Buehler T, Kreis R, Decombaz J (2012). Effect of two beta-alanine dosing protocols on muscle carnosine synthesis and washout. Amino Acids.

[20] Derave W, Ozdemir MS, Harris RC, Pottier A, Reyngoudt H, Koppo K (2007). beta-Alanine supplementation augments muscle carnosine content and attenuates fatigue during repeated isokinetic contraction bouts in trained sprinters. J Appl Physiol (1985).

[21] Bex T, Chung W, Baguet A, Stegen S, Stautemas J, Achten E (2014). Muscle carnosine loading by beta-alanine supplementation is more pronounced in trained vs. untrained muscles. J Appl Physiol (1985).

[22] Stout JR, Cramer JT, Zoeller RF, Torok D, Costa P, Hoffman JR (2007). Effects of beta-alanine supplementation on the onset of neuromuscular fatigue and ventilatory threshold in women. Amino Acids.

[23] Stegen S, Bex T, Vervaet C, Vanhee L, Achten E, Derave W (2014). beta-Alanine dose for maintaining moderately elevated muscle carnosine levels. Med Sci Sports Exerc.

[24] Stout JR, Graves BS, Smith AE, Hartman MJ, Cramer JT, Beck TW (2008). The effect of beta-alanine supplementation on neuromuscular fatigue in elderly (55–92 Years): a double-blind randomized study. J Int Soc Sports Nutr.

[25] Sale C, Saunders B, Harris RC (2010). Effect of beta-alanine supplementation on muscle carnosine concentrations and exercise performance. Amino Acids.

[26] Jackson MC, Kucera CM, Lenney JF (1991). Purification and properties of human serum carnosinase. Clin Chim Acta.

[27] Gardner ML, Illingworth KM, Kelleher J, Wood D (1991). Intestinal absorption of the intact peptide carnosine in man, and comparison with intestinal permeability to lactulose. J Physiol.

[28] Severin SE, Kirzon MV, Kaftanova TM (1953). [Effect of carnosine and anserine on action of isolated frog muscles]. Dokl Akad Nauk SSSR.

[29] Tanokura M, Tasumi M, Miyazawa T (1976). 1H nuclear magnetic resonance studies of histidine-containing di- and tripeptides. Estimation of the effects of charged groups on the pKa value of the imidazole ring. Biopolymers.

[30] Suzuki Y, Nakao T, Maemura H, Sato M, Kamahara K, Morimatsu F (2006). Carnosine and anserine ingestion enhances contribution of nonbicarbonate buffering. Med Sci Sports Exerc.

[31] Davey CL (1960). The significance of carnosine and anserine in striated skeletal muscle. Arch Biochem Biophys.

[32] Baguet A, Koppo K, Pottier A, Derave W (2010). Beta-alanine supplementation reduces acidosis but not oxygen uptake response during high-intensity cycling exercise. Eur J Appl Physiol.

[33] Powers SK, Jackson MJ (2008). Exercise-induced oxidative stress: cellular mechanisms and impact on muscle force production. Physiol Rev.

[34] Bailey DM, Davies B, Young IS, Hullin DA, Seddon PS (2001). A potential role for free radical-mediated skeletal muscle soreness in the pathophysiology of acute mountain sickness. Aviat Space Environ Med.

[35] Venditti P, Di Meo S (1997). Effect of training on antioxidant capacity, tissue damage, and endurance of adult male rats. Int J Sports Med.

[36] Klebanov GI, Teselkin Yu O, Babenkova IV, Lyubitsky OB, Rebrova O, Boldyrev AA (1998). Effect of carnosine and its components on free-radical reactions. Membr Cell Biol.

[37] Kohen R, Yamamoto Y, Cundy KC, Ames BN (1988). Antioxidant activity of carnosine, homocarnosine, and anserine present in muscle and brain. Proc Natl Acad Sci U S A.

[38] Hoffman J, Ratamess NA, Ross R, Kang J, Magrelli J, Neese K (2008). Beta-alanine and the hormonal response to exercise. Int J Sports Med.

[39] Harris RC, Jones GA, Kim HJ, Kim CK, Price KA, Wise JA (2009). Changes in muscle carnosine of subjects with 4 weeks of supplementation with a controlled relase formulation of beta-alanine (CarnoSyn), and for 6 weeks post (Abstract). FASEB J.

[40] Stellingwerff T, Decombaz J, Harris RC, Boesch C (2012). Optimizing human in vivo dosing and delivery of beta-alanine supplements for muscle carnosine synthesis. Amino Acids.

[41] Stegen S, Blancquaert L, Everaert I, Bex T, Taes Y, Calders P (2013). Meal and beta-alanine coingestion enhances muscle carnosine loading. Med Sci Sports Exerc.

[42] Hobson RM, Saunders B, Ball G, Harris RC, Sale C (2012). Effects of beta-alanine supplementation on exercise performance: a meta-analysis. Amino Acids.

[43] Shinohara T, Harada M, Ogi K, Maruyama M, Fujii R, Tanaka H (2004). Identification of a G protein-coupled receptor specifically responsive to beta-alanine. J Biol Chem.

[44] Crozier RA, Ajit SK, Kaftan EJ, Pausch MH (2007). MrgD activation inhibits KCNQ/M-currents and contributes to enhanced neuronal excitability. J Neurosci.

[45] Macphee S, Weaver IN, Weaver DF (2013). An Evaluation of Interindividual Responses to the Orally Administered Neurotransmitter beta-Alanine. J Amino Acids.

[46] Murakami T, Furuse M (2010). The impact of taurine- and beta-alanine-supplemented diets on behavioral and neurochemical parameters in mice: antidepressant versus anxiolytic-like effects. Amino Acids.

[47] Dawson R, Biasetti M, Messina S, Dominy J (2002). The cytoprotective role of taurine in exercise-induced muscle injury. Amino Acids.

[48] Cramer JT. Creatine Supplementation in Endurance Sports. In: Stout JR, Antonio J, Kalman D, editors. Essentials of Creatine in Sports and Health. Totowa, New Jersey: Humana Press; 2008. p. 45–99.

[49] Shrier I (2004). Does stretching improve performance? A systematic and critical review of the literature. Clin J Sport Med.

[50] Culbertson JY, Kreider RB, Greenwood M, Cooke M (2010). Effects of beta-alanine on muscle carnosine and exercise performance: a review of the current literature. Nutrients.

[51] Skulachev VP (2000). Biological role of carnosine in the functioning of excitable tissues. Centenary of Gulewitsch's discovery. Biochemistry (Mosc).

[52] Beaver WL, Wasserman K, Whipp BJ (1986). Bicarbonate buffering of lactic acid generated during exercise. J Appl Physiol (1985).

[53] Sweeney KM, Wright GA, Glenn Brice A, Doberstein ST (2010). The effect of beta-alanine supplementation on power performance during repeated sprint activity. J Strength Cond Res.

[54] Ghiasvand R, Askari G, Malekzadeh J, Hajishafiee M, Daneshvar P, Akbari F (2012). Effects of Six Weeks of beta-alanine Administration on VO(2) max, Time to Exhaustion and Lactate Concentrations in Physical Education Students. Int J Prev Med.

[55] Jagim AR, Wright GA, Brice AG, Doberstein ST (2013). Effects of beta-alanine supplementation on sprint endurance. J Strength Cond Res.

[56] Smith-Ryan AE, Fukuda DH, Stout JR, Kendall KL (2012). High-velocity intermittent running: effects of beta-alanine supplementation. J Strength Cond Res.

[57] Van Thienen R, Van Proeyen K, Vanden Eynde B, Puype J, Lefere T, Hespel P (2009). Beta-alanine improves sprint performance in endurance cycling. Med Sci Sports Exerc.

[58] Zoeller RF, Stout JR, O'Kroy JA, Torok DJ, Mielke M (2007). Effects of 28 days of beta-alanine and creatine monohydrate supplementation on aerobic power, ventilatory and lactate thresholds, and time to exhaustion. Amino Acids.

[59] Smith AE, Walter AA, Graef JL, Kendall KL, Moon JR, Lockwood CM (2009). Effects of beta-alanine supplementation and high-intensity interval training on endurance performance and body composition in men; a double-blind trial. J Int Soc Sports Nutr.

[60] Smith-Ryan AE, Woessner MN, Melvin MN, Wingfield HL, Hackney AC (2014). The effects of beta-alanine supplementation on physical working capacity at heart rate threshold. Clin Physiol Funct Imaging.

[61] Baguet A, Bourgois J, Vanhee L, Achten E, Derave W (2010). Important role of muscle carnosine in rowing performance. J Appl Physiol (1985).

[62] de Salles PV, Roschel H, de Jesus F, Sale C, Harris RC, Solis MY (2013). The ergogenic effect of beta-alanine combined with sodium bicarbonate on high-intensity swimming performance. Appl Physiol Nutr Metab.

[63] Ducker KJ, Dawson B, Wallman KE (2013). Effect of beta-alanine supplementation on 800-m running performance. Int J Sport Nutr Exerc Metab.

[64] Ducker KJ, Dawson B, Wallman KE (2013). Effect of beta-alanine supplementation on 2000-m rowing-ergometer performance. Int J Sport Nutr Exerc Metab.

[65] Hobson RM, Harris RC, Martin D, Smith P, Macklin B, Gualano B (2013). Effect of Beta-Alanine With and Without Sodium Bicarbonate on 2,000-m Rowing Performance. Int J Sport Nutr Exerc Metab.

[66] Kern BD, Robinson TL (2011). Effects of beta-alanine supplementation on performance and body composition in collegiate wrestlers and football players. J Strength Cond Res.

[67] Chung W, Shaw G, Anderson ME, Pyne DB, Saunders PU, Bishop DJ (2012). Effect of 10 week beta-alanine supplementation on competition and training performance in elite swimmers. Nutrients.

[68] Sale C, Saunders B, Hudson S, Wise JA, Harris RC, Sunderland CD (2011). Effect of beta-alanine plus sodium bicarbonate on high-intensity cycling capacity. Med Sci Sports Exerc.

[69] Danaher J, Gerber T, Wellard RM, Stathis CG (2014). The effect of beta-alanine and NaHCO3 co-ingestion on buffering capacity and exercise performance with high-intensity exercise in healthy males. Eur J Appl Physiol.

[70] Chung W, Baguet A, Bex T, Bishop DJ, Derave W (2014). Doubling of muscle carnosine concentration does not improve laboratory 1-h cycling time-trial performance. Int J Sport Nutr Exerc Metab.

[71] Stout JR, Cramer JT, Mielke M, O'Kroy J, Torok DJ, Zoeller RF (2006). Effects of twenty-eight days of beta-alanine and creatine monohydrate supplementation on the physical working capacity at neuromuscular fatigue threshold. J Strength Cond Res.

[72] Smith AE, Moon JR, Kendall KL, Graef JL, Lockwood CM, Walter AA (2009). The effects of beta-alanine supplementation and high-intensity interval training on neuromuscular fatigue and muscle function. Eur J Appl Physiol.

[73] McCormack WP, Stout JR, Emerson NS, Scanlon TC, Warren AM, Wells AJ (2013). Oral nutritional supplement fortified with beta-alanine improves physical working capacity in older adults: a randomized, placebo-controlled study. Exp Gerontol.

[74] Hoffman JR, Ratamess NA, Faigenbaum AD, Ross R, Kang J, Stout JR (2008). Short-duration beta-alanine supplementation increases training volume and reduces subjective feelings of fatigue in college football players. Nutr Res.

[75] Sale C, Hill CA, Ponte J, Harris RC (2012). beta-alanine supplementation improves isometric endurance of the knee extensor muscles. J Int Soc Sports Nutr.

[76] Hoffman J, Ratamess N, Kang J, Mangine G, Faigenbaum A, Stout J (2006). Effect of creatine and beta-alanine supplementation on performance and endocrine responses in strength/power athletes. Int J Sport Nutr Exerc Metab.

[77] Hoffman JR, Landau G, Stout JR, Dabora M, Moran DS, Sharvit N (2014). beta-alanine supplementation improves tactical performance but not cognitive function in combat soldiers. J Int Soc Sports Nutr.

[78] Ko R, Low Dog T, Gorecki DK, Cantilena LR, Costello RB, Evans WJ (2014). Evidence-based evaluation of potential benefits and safety of beta-alanine supplementation for military personnel. Nutr Rev.

[79] Hoffman JR, Landau G, Stout JR, Hoffman MW, Shavit N, Rosen P (2015). beta-Alanine ingestion increases muscle carnosine content and combat specific performance in soldiers. Amino Acids.

[80] Solis MY, Cooper S, Hobson RM, Artioli GG, Otaduy MC, Roschel H (2015). Effects of Beta-alanine supplementation on brain homocarnosine/carnosine signal and cognitive function: an exploratory study. PLoS One.

[81] Peart DJ, Siegler JC, Vince RV (2012). Practical recommendations for coaches and athletes: a meta-analysis of sodium bicarbonate use for athletic performance. J Strength Cond Res.

[82] Tobias G, Benatti FB, de Salles PV, Roschel H, Gualano B, Sale C (2013). Additive effects of beta-alanine and sodium bicarbonate on upper-body intermittent performance. Amino Acids.

[83] Mero AA, Hirvonen P, Saarela J, Hulmi JJ, Hoffman JR, Stout JR (2013). Effect of sodium bicarbonate and beta-alanine supplementation on maximal sprint swimming. J Int Soc Sports Nutr.

[84] Ducker KJ, Dawson B, Wallman KE (2013). Effect of Beta alanine and sodium bicarbonate supplementation on repeated-sprint performance. J Strength Cond Res.

[85] Saunders B, Sale C, Harris RC, Sunderland C (2014). Effect of sodium bicarbonate and Beta-alanine on repeated sprints during intermittent exercise performed in hypoxia. Int J Sport Nutr Exerc Metab.

[86] Bellinger PM, Howe ST, Shing CM, Fell JW (2012). Effect of combined beta-alanine and sodium bicarbonate supplementation on cycling performance. Med Sci Sports Exerc.

[87] Carr AJ, Slater GJ, Gore CJ, Dawson B, Burke LM (2011). Effect of sodium bicarbonate on [HCO3-], pH, and gastrointestinal symptoms. Int J Sport Nutr Exerc Metab.

[88] Branch JD (2003). Effect of creatine supplementation on body composition and performance: a meta-analysis. Int J Sport Nutr Exerc Metab.

[89] Harris RC, Hill C, Wise JA (2003). Effect of combined beta-alanine and creatine monohydrate supplementation on exercise performance (Abstract). Med Sci Sports Exerc.

[90] Kresta JY, Oliver JM, Jagim AR, Fluckey J, Riechman S, Kelly K (2014). Effects of 28 days of beta-alanine and creatine supplementation on muscle carnosine, body composition and exercise performance in recreationally active females. J Int Soc Sports Nutr.

[91] Walsh AL, Gonzalez AM, Ratamess NA, Kang J, Hoffman JR (2010). Improved time to exhaustion following ingestion of the energy drink Amino Impact. J Int Soc Sports Nutr.

[92] Spradley BD, Crowley KR, Tai CY, Kendall KL, Fukuda DH, Esposito EN (2012). Ingesting a pre-workout supplement containing caffeine, B-vitamins, amino acids, creatine, and beta-alanine before exercise delays fatigue while improving reaction time and muscular endurance. Nutr Metab (Lond).

[93] Spillane M, Schwarz N, Leddy S, Correa T, Minter M, Longoria V (2011). Effects of 28 days of resistance exercise while consuming commercially available pre- and post-workout supplements, NO-Shotgun(R) and NO-Synthesize(R) on body composition, muscle strength and mass, markers of protein synthesis, and clinical safety markers in males. Nutr Metab (Lond).

[94] Shelmadine B, Cooke M, Buford T, Hudson G, Redd L, Leutholtz B (2009). Effects of 28 days of resistance exercise and consuming a commercially available pre-workout supplement, NO-Shotgun(R), on body composition, muscle strength and mass, markers of satellite cell activation, and clinical safety markers in males. J Int Soc Sports Nutr.

[95] Ormsbee MJ, Thomas DD, Mandler WK, Ward EG, Kinsey AW, Panton LB (2013). The effects of pre- and post-exercise consumption of multi-ingredient performance supplements on cardiovascular health and body fat in trained men after six weeks of resistance training: a stratified, randomized, double-blind study. Nutr Metab (Lond).

[96] Ormsbee MJ, Mandler WK, Thomas DD, Ward EG, Kinsey AW, Simonavice E (2012). The effects of six weeks of supplementation with multi-ingredient performance supplements and resistance training on anabolic hormones, body composition, strength, and power in resistance-trained men. J Int Soc Sports Nutr.

[97] Kendall KL, Moon JR, Fairman CM, Spradley BD, Tai CY, Falcone PH (2014). Ingesting a preworkout supplement containing caffeine, creatine, beta-alanine, amino acids, and B vitamins for 28 days is both safe and efficacious in recreationally active men. Nutr Res.

[98] Gonzalez AM, Walsh AL, Ratamess NA, Kang J, Hoffman JR (2011). Effect of a pre-workout energy supplement on acute multi-joint resistance exercise. J Sports Sci Med.

[99] Outlaw JJ, Wilborn CD, Smith-Ryan AE, Hayward SE, Urbina SL, Taylor LW (2014). Acute effects of a commercially-available pre-workout supplement on markers of training: a double-blind study. J Int Soc Sports Nutr.

[100] Hipkiss AR (2005). Glycation, ageing and carnosine: are carnivorous diets beneficial?. Mech Ageing Dev.

[101] Hipkiss AR, Cartwright SP, Bromley C, Gross SR, Bill RM (2013). Carnosine: can understanding its actions on energy metabolism and protein homeostasis inform its therapeutic potential?. Chem Cent J.

[102] Hipkiss AR, Brownson C, Carrier MJ (2001). Carnosine, the anti-ageing, anti-oxidant dipeptide, may react with protein carbonyl groups. Mech Ageing Dev.

[103] Hipkiss AR, Michaelis J, Syrris P (1995). Non-enzymatic glycosylation of the dipeptide L-carnosine, a potential anti-protein-cross-linking agent. FEBS Lett.

[104] Decker EA, Crum AD, Calvert JT (1992). Differences in the antioxidant mechanism of carnosine in the presence of copper and iron. J Agric Food Chem.

[105] Decker EA, Ivanov V, Zhu BZ, Frei B (2001). Inhibition of low-density lipoprotein oxidation by carnosine histidine. J Agric Food Chem.

[106] Gariballa SE, Sinclair AJ (2000). Carnosine: physiological properties and therapeutic potential. Age Ageing.

[107] Smith AE, Stout JR, Kendall KL, Fukuda DH, Cramer JT (2012). Exercise-induced oxidative stress: the effects of beta-alanine supplementation in women. Amino Acids.

[108] Smith-Ryan AE, Fukuda DH, Stout JR, Kendall KL (2014). The influence of beta-alanine supplementation on markers of exercise-induced oxidative stress. Appl Physiol Nutr Metab.

[109] Boldyrev A, Kurella E, Stvolinski S (1994). Biological role of carnosine metabolism in excitable tissues: speculations and facts. Pathophysiology.

[110] Hoffman JR, Ostfeld I, Stout JR, Harris RC, Kaplan Z, Cohen H (2015). beta-Alanine supplemented diets enhance behavioral resilience to stress exposure in an animal model of PTSD. Amino Acids.

[111] Dutka TL, Lamb GD (2004). Effect of carnosine on excitation-contraction coupling in mechanically-skinned rat skeletal muscle. J Muscle Res Cell Motil.

[112] Lamont C, Miller DJ (1992). Calcium sensitizing action of carnosine and other endogenous imidazoles in chemically skinned striated muscle. J Physiol.

[113] Hannah R, Stannard RL, Minshull C, Artioli GG, Harris RC, Sale C (2015). beta-Alanine supplementation enhances human skeletal muscle relaxation speed but not force production capacity. J Appl Physiol (1985).

